# Nollmotzite, Mg[U^V^(U^VI^O_2_)_2_O_4_F_3_]·4H_2_O, the first natural uranium oxide containing fluorine

**DOI:** 10.1107/S2052520618007321

**Published:** 2018-06-28

**Authors:** Jakub Plášil, Anthony R. Kampf, Radek Škoda, Jiří Čejka

**Affiliations:** a Institute of Physics ASCR, v.v.i., Na Slovance 2, Praha 8, 18221, Czech Republic; b Mineral Sciences Department, Natural History Museum of Los Angeles County, 900 Exposition Boulevard, Los Angeles, CA 90007, USA; c Department of Geological Sciences, Faculty of Science, Masaryk University, Kotlářská 2, Brno, 61137, Czech Republic; d Department of Mineralogy and Petrology, National Museum, Cirkusová 1740, Prague 9, 19300, Czech Republic

**Keywords:** nollmotzite, new mineral, uranium oxide fluoride, pentavalent uranium

## Abstract

Nollmotzite (IMA2017-100), Mg[U^V^(U^VI^O_2_)_2_F_3_O_4_](H_2_O)_4_, is a new uranium oxide fluoride mineral found in the Clara mine, Black Forest Mountains, Germany. Electron microprobe analysis provided the empirical formula (Mg_1.06_Cu_0.02_)_Σ1.08_[U^V^(U^VI^O_2_)_2_O_3.85_F_3.15_][(H_2_O)_3.69_(OH)_0.31_]_Σ4.00_ based on three U and 15 O + F atoms per formula unit. Nollmotzite is monoclinic, space group *Cm*, with *a* = 7.1015 (12) Å, *b* = 11.7489 (17) Å, *c* = 8.1954 (14) Å, β = 98.087 (14)°, *V* = 676.98 (19) Å^3^ and *Z* = 2. The crystal structure [twinned by reticular merohedry; refined to *R* = 0.0369 with GoF = 1.09 for 1527 unique observed reflections, *I* > 3σ(*I*)] is based upon [U^V^(U^VI^O_2_)_2_F_3_O_4_]^2–^ sheets of β-U_3_O_8_ topology and contains an interlayer with MgF_2_(H_2_O)_4_ octahedra. Adjacent sheets are linked through F–Mg–F bonds, as well as *via* hydrogen bonds. The presence of fluorine and pentavalent uranium in the structure of nollmotzite has potentially important implications for the safe disposal of nuclear waste.

## Abstract 2

The twinned structure of the new uranium oxide mineral was refined from X-ray diffraction data and was found to contain fluorine and pentavalent uranium. The presence of pentavalent uranium is indicative of the reducing conditions under which the mineral formed. Nollmotzite is the first naturally occurring uranium oxide mineral that contains fluorine.

## 1. Introduction   

Uranium oxides, especially those containing U^VI^ as the uranyl (UO_2_^2+^) ion, are important products of supergene weathering of primary U^IV^ minerals, predominantly uraninite, UO_2_. Uranium dioxide, both in nuclear fuel and uraninite, readily alters in the presence of water and oxygen resulting in the forma­tion of uranyl-oxide hy­droxy-hydrate minerals (UOH) (Finch & Ewing, 1992 ▸; Wronkiewicz *et al.*, 1992 ▸, 1996 ▸; Janeczek *et al.*, 1996 ▸, and others). UOH minerals are among the first phases to form during the oxidation–hydration weathering of UO_2_ (Finch & Ewing, 1992 ▸; Finch *et al.*, 1996 ▸; Schindler & Hawthorne, 2004 ▸; Krivovichev & Plášil, 2013 ▸; Plášil, 2014 ▸). Because of their importance for nuclear waste disposal and the environmental chemistry of uranium in general (see *e.g.* O’Hare *et al.*, 1988 ▸; Finch & Murakami, 1999 ▸; Klingensmith *et al.*, 2007 ▸; Kubatko *et al.* 2006 ▸; Maher *et al.*, 2013 ▸), stud­ies describing their structures and physical–chemical properties, such as solubility and thermodynamic stability, are numer­ous. Herein, we provide a description of the new mineral nollmotzite, which is the first naturally occurring uranium oxide that contains fluorine. It also is noteworthy for containing uranium as both U^V^ and U^VI^.

The name nollmotzite honors two German mineral collectors who discovered this new mineral; the name nollmotzite combines the first four letters of their surnames: Markus Noller (born 16.05.1977) and Reinhard Motzigemba (born 14.11.1952). Both the name and the new mineral were approved by the Commission on New Minerals, Nomenclature and Classification of the International Mineralogical Association (proposal IMA2017-100). The description is based on three cotype specimens deposited in the collections of the Natural History Museum of Los Angeles County, CA, USA, with catalog numbers 66647, 66648 and 66649.

## 2. Methodology   

### 2.1. Sample   

**Figure 1:**
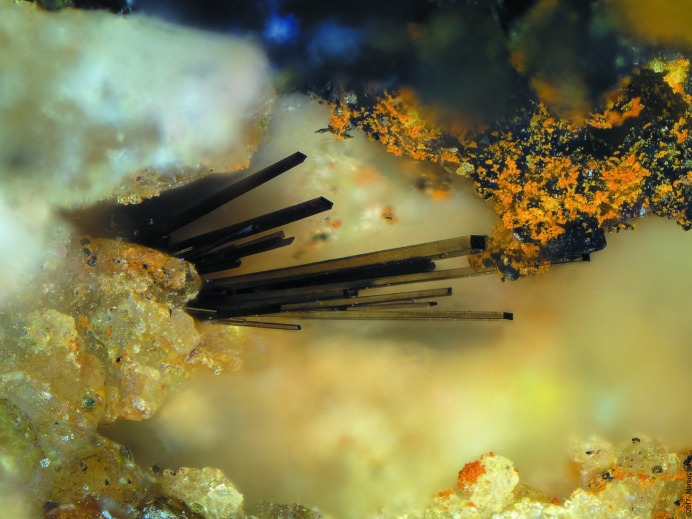
Nollmotzite crystals with typical chisel-like terminations in quartz–fluorite gangue. Field of view *ca* 1.2 mm across (photo by M. Noller).

Nollmotzite was found in June 2016 on the dump of the famous Clara mine in the Black Forest Mountains, Baden-Württemberg, Germany. Nollmotzite grows in cavities in quartz gangue with abundant dark-violet (nearly black) fluorite (so-called stinkspath) and barite. It forms thin prisms, elongated on [010], with chisel-like terminations (Figs. 1 ▸ and 2 ▸) up to about 0.3 mm in length. Crystals exhibit the forms (100), 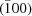, (001), 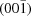, (120) and 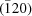. They are deep-violet–brown in color and are transparent with vitreous luster. Nollmotzite is non-fluorescent under longwave and shortwave ultraviolet radiation. Crystals are brittle, with a perfect cleavage on {001}. Examination by polarized light microscopy shows that nollmotzite is strongly pleochroic, *X* = colorless, *Y* = red–brown, *Z* = deep violet (*X* << *Y* < *Z*). The optical orientation is *X* ≃ c*, *Y* = **b**, *Z* ≃ **a** (*X*^**c** ≃ 9° in obtuse β). Crystals are optically biaxial (−), with α = 1.615 (3), β = 1.750 (5), γ = 1.765 (5) (white light), *n*_average_ = 1.710, 2*V*_meas_ = 37 (1)° from extinction data analyzed using *EXCALIBRW* (Gunter *et al.*, 2004 ▸), 2*V*_calc_ = 34.6°, dispersion is strong, *r* > *v*.

### 2.2. Electron microprobe   

The chemical composition of nollmotzite was determined using a Cameca SX100 electron microprobe (WDS mode, 15 kV, 4 nA, 5 µm beam diameter). Because insufficient material was available for a direct determination of H_2_O, it has been calculated by stoichiometry on the basis of three U and 15 O + F atoms per formula unit (apfu) in accord with the crystal structure determination. No other elements with atomic numbers higher than eight were observed. Analytical data are given in Table 1 ▸. The empirical formula is (Mg_1.06_Cu_0.02_)_Σ1.08_[U^V^(U^VI^O_2_)_2_O_3.85_F_3.15_][(H_2_O)_3.69_(OH)_0.31_]_Σ4.00_ (note that the OH is for charge balance and does not imply that some H_2_O sites are OH).

### 2.3. Raman spectroscopy   

**Figure 3:**
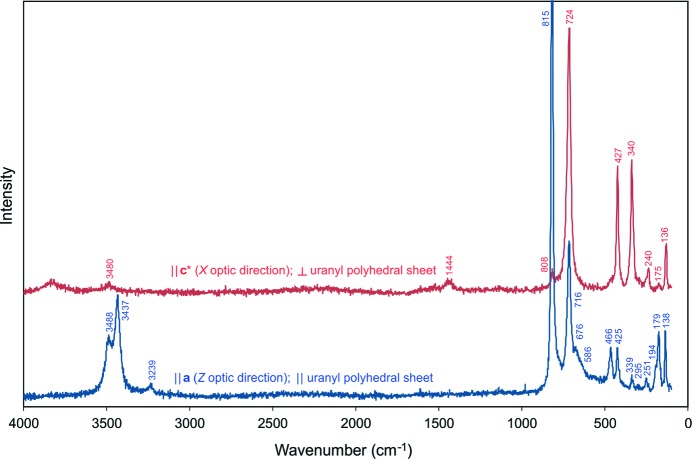
Raman spectra of nollmotzite collected with a 532 nm laser.

Raman spectra of nollmotzite were recorded on a Horiba XploRA Plus spectrometer using a 532 nm diode laser. The spectra were recorded in two orientations: || c* (X optic direction), ⊥ to the sheet of U polyhedra; and || **a** (Z optic direction), || to the sheet of U polyhedra (Fig. 3 ▸).

### 2.4. X-ray diffraction   

#### 2.4.1. Powder diffraction   

X-ray powder diffraction data were recorded using a Rigaku R-Axis Rapid II curved imaging plate microdiffractometer with monochromated Mo *K*α. A Gandolfi-like motion on the φ and ω axes was used to randomize the sample. Observed *d* values and intensities were derived by profile fitting using *JADE 2010* (https://materialsdata.com/prodjd.html/) software. Data are given in the Table S1. Unit-cell parameters refined from the powder data using *JADE 2010* with whole pattern fitting are as follows: *a* = 7.117 (6) Å, *b* = 11.786 (7) Å, *c* = 8.203 (6) Å, β = 98.14 (2)°, with *V* = 681.1 (9) Å^3^ and *Z* = 2.

### 2.5. Single-crystal diffraction   

For the single-crystal diffraction experiment, the crystal was selected under an polarized light microscope and mounted on a glass fiber. The diffraction experiment (see Table 2 ▸ for details) was performed at room temperature with a Rigaku SuperNova single-crystal diffractometer with an Atlas S2 CCD detector, using mirror monochromated Mo *K*α radiation from a microfocus X-ray tube. According to the single-crystal X-ray experiment, nollmotzite is monoclinic, with space group *Cm*. Corrections for background, Lorentz, and polarization effects, as well as absorption correction were applied during data reduction in the *CrysAlis* (Rigaku Oxford Diffraction, 2017 ▸) package. Two datasets, representing two twin domains (characterized by distinct orientation matrix and related by the twin law) were produced by the data reduction (see §2.6). The bond-valence sums were calculated following the procedure of Brown (2002 ▸), and utilizing bond-valence parameters taken from Burns *et al.* (1997*a* ▸) and Gagné & Hawthorne (2015 ▸).

### 2.6. Structure solution and refinement of the twinned structure   

**Figure 4:**
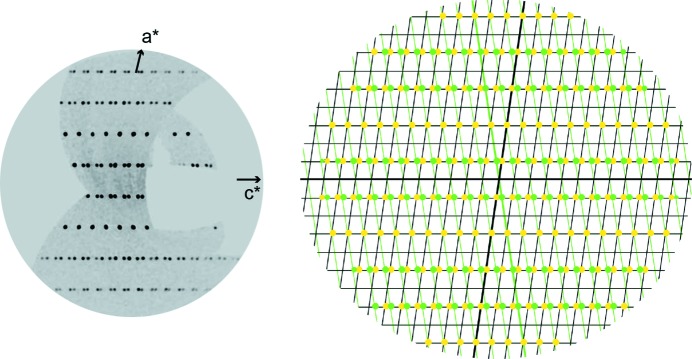
A twinned diffraction pattern of nollmotzite. A precession-like image (left) of reciprocal space, reconstructed with the UNWARP procedure of the *CrysAlis* software, *h*3*l* plane and (right) the same diffraction pattern calculated using *Jana2006* from the structure model determined here. Yellow spots belong to the first (larger) domain (with the corresponding lattice shown with black lines), while green spots belong to the second (weaker) twin domain (with the corresponding lattice drawn with the green lines).

The structure of nollmotzite was solved from diffraction data using the charge-flipping algorithm of the program *SHELXT* (Sheldrick, 2015 ▸), and subsequently, it was refined using the *Jana2006* (Petříček *et al.*, 2014 ▸) program, based on *F* ^2^. The output from the *SHELXT* program suggested for monoclinic space group *Cm*, which was later confirmed by the refinement. A quick test in *Jana2006* revealed the possible presence of a twin by reticular (pseudo)merohedry (Petříček *et al.*, 2016 ▸), which can be indexed in the orthorhombic super-cell. Careful inspection of diffraction frames and reconstruction of the reciprocal space of nollmotzite [UNWARP procedure in the *CrysAlis* (Rigaku Oxford Diffraction, 2017 ▸) software] confirmed the presence of diffractions of the second twin domain. The twin is by reflection on (100) leading to a complete overlap of reflections with *h + k* = 2*n*, and non-overlapping reflections occur at a third of the real c* parameter (Fig. 4 ▸). Taking the twinning into account, the refinement converged smoothly to more acceptable residuals (*R* = 3.69% with GoF = 1.09 for 1527 unique observed reflections) (Table 2 ▸). During final cycles of the refinement, the O2 and O6 atoms were restricted to have the same atomic displacement parameters, because the O2 atom returned a low atomic displacement parameter value (*U*_iso_ = 0.003 Å^2^). We cannot exclude the possibility that this site is partially occupied by F; however, a more probable explanation is that the low atomic displacement parameter is a relic resulting from an imperfect correction for the high absorption. Hydrogen atom positions were not determined due to the weak X-ray scattering factor of hydrogen and the predominance of uranium in the difference Fourier density maps. Final atom coordinates and displacement parameters are given in Tables S2 and S3, selected interatomic distances in Table 3 ▸ and bond-valence sums in Table 4 ▸.

### 2.7. Voronoi–Dirichlet polyhedra calculations   

According to the stereoatomic model of crystals (*e.g.* Blatov *et al.*, 1995 ▸, 1999 ▸), their structure is considered as being a partition of the three-dimensional space, where geometrical images of atoms are their Voronoi–Dirichlet polyhedra (VDP). For details of the application of VDP to the crystal chemistry of uranium, we refer to the paper by Serezhkin (2007 ▸). The number of faces, the form and volume (*V*_VDP_) of the VDP are uniquely determined by the exact position of the atom in the particular structure. The linear parameters that characterize dimensions of a particular atom in the structure are the radius of the sphere, *R*_SD_ (in Å) and its volume equal to *V*_VDP_ (in Å^3^). Each face of VDP corresponds to a particular kind of interatomic interaction (*i.e.* bond). There are several parameters describing distortion of the atomic coordination in the VDP (Blatov *et al.*, 1995 ▸; Blatov & Serezhkin, 2000 ▸); among them, the second moment of inertia, *G*_3_, describing the deviation of the VDP from an ideal sphere and characterizing the uniformity of distribution of the atoms around the centroid atom (here U). For an ideal sphere *G*_3_ = 0.077, whereas for an ideal heteroatomic *AX*_6_ octahedron (which corresponds to a cubic VD polyhedron), *G*_3_ = 0.0833 (Blatov & Serezhkin, 2000 ▸). The properties of Voronoi–Dirichlet polyhedra were calculated by the program *Topos* (Blatov *et al.*, 2014 ▸).

## 3. Results   

### 3.1. Raman spectroscopy   

In the region of O—H stretching vibrations, clear differences between two spectral orientations occur. In the spectrum || **a**, there is a pronounced two-component polarized band occurring at 3450 cm^−1^ (one at ∼3488 cm^−1^ and a stronger band at 3437 cm^−1^) and also an additional weak band at 3239 cm^−1^. These bands are related to the symmetric O—H stretching vibrations of the water molecules bonded in the structure by hydrogen bonds of distinct strengths. The hydrogen bonds in the studied crystal correspond to O⋯O distances in the range from 2.81 (3) to 2.90 (4) Å, fitting well with the correlation given by Libowitzky (1999 ▸). As expected, only a very weak band was observed at ∼3480 cm^−1^ in the spectrum || c*. We assume it is due to the fact that there are fewer hydrogen bonds from the interlayer to the structure sheets; most of the hydrogen bonds are more or less parallel to the structural sheets. There is no band observed related to H—O—H bending vibrations, which is not unusual for Raman spectroscopy. A weak band at 1444 cm^−1^, observed in the spectrum || c*, may be assigned to an overtone or a combination band. A very strong band observed at 815 cm^−1^ in the spectrum || **a** is attributed to the ν_1_ (UO_2_)^2+^ symmetric stretching vibration. According to Bartlett & Cooney (1989 ▸), an approximate U—O bond length of 1.8 Å is in line with the bond length obtained from the structure [*c.f.* U2—O = 1.77 (2) and 1.81 (2) Å]. These values are also in line with uranyl U—O lengths in UO_7_ pentagonal bipyramids given by Lussier *et al.* (2016 ▸). A very weak band at 808 cm^−1^ in the spectrum || c* may be attributed to the same vibration as the symmetric stretching mode should be highly polarizable. A band of high intensity at 716 cm^−1^ in the spectrum || **a**, and at 724 cm^−1^ in the spectrum || c*, is most probably connected with the ν_1_ U—F symmetric stretching vibration. The shift towards the lower energy is due to the higher mass of F compared with that of O. Shoulders at 676 and 586 cm^−1^ in the spectrum || **a** are assigned to libration modes of molecular H_2_O (Lutz, 1988 ▸). Bands of medium intensity at 466 and 425 cm^−1^, plus the weak band at 339 cm^−1^ (|| **a**), and strong bands at 427 and 340 cm^−1^ (|| c*) are connected with the ν(U—O,F_ligand_) vibrations. Weak bands at 295 cm^−1^ and 251 cm^−1^ (|| **a**) and a weak band at 240 cm^−1^ (|| c*) are assigned to the ν_2_ (δ) (UO_2_)^2+^ doubly degenerate bending vibrations. A band of medium intensity at 179 cm^−1^ (|| **a**) and a very weak band at 175 cm^−1^ (|| c*) may be assigned to the δ F–U–F bending vibrations; a shoulder- and a low-intensity band at 194 and 138 cm^−1^, respectively (|| **a**) and a weak band at 136 cm^−1^ (|| c*) are connected with translations and rotations of (UO_2_)^2+^ (Dothée & Camelot, 1982 ▸; Čejka *et al.*, 1998 ▸).

### 3.2. Crystal structure   

**Figure 5:**
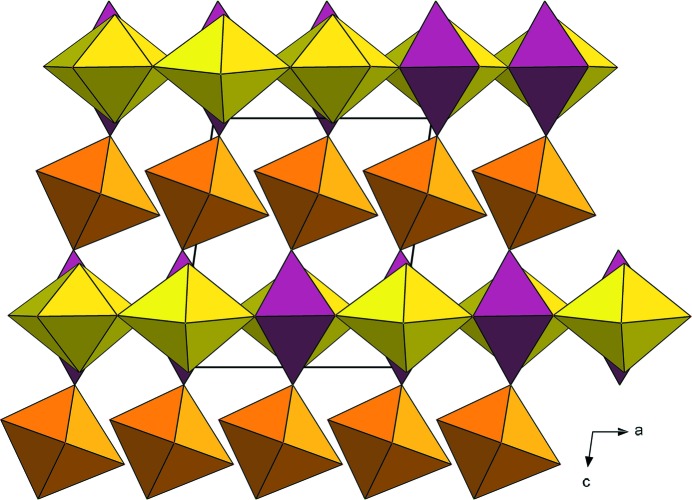
Crystal structure of nollmotzite viewed along **b**. The sheets of U polyhedra (yellow and violet) alternate interlayer with Mg(H_2_O)_4_ octahedra (orange).

**Figure 6:**
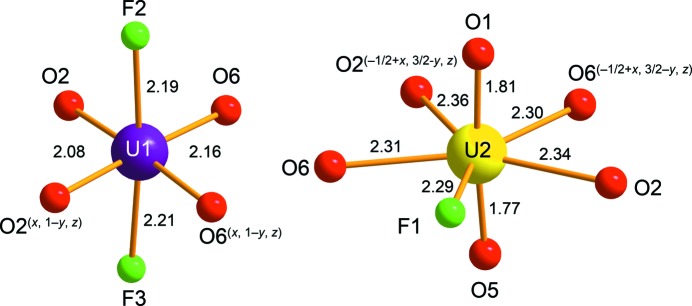
Coordination of U atoms in the structure of nollmotzite; violet U1 is U^V^ and yellow U2 is U^VI^. Bond lengths are given in Å.

**Figure 7:**
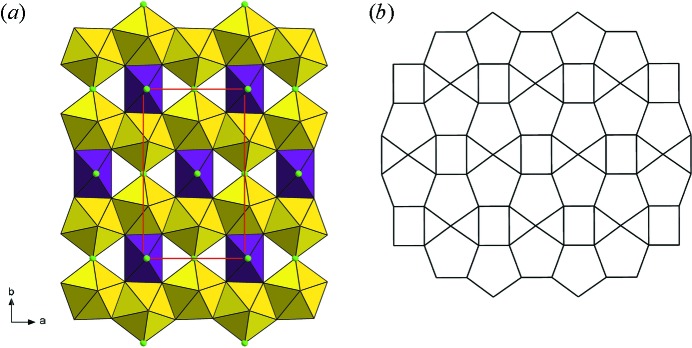
(*a*) The [U^V^(U^VI^O_2_)_2_O_4_F_3_]^2−^ sheet in the structure of nollmotzite. The F sites are displayed in green, U^V^ polyhedra (U1) are in violet, U^VI^ polyhedra (U2) are in yellow; unit-cell edges outlined with a solid red line. (*b*) Its respective topology (β-U_3_O_8_ type).

The structure of nollmotzite (Table S2) contains two U, one Mg, three F and seven O sites (including three O sites of the molecular H_2_O) (Fig. 5 ▸). The U1 site (Wyckoff notation 2*a*, site symmetry *m*) is linked to six ligands, four O (O2 2×; O6 2×) and two F atoms (F2 and F3), while two F atoms are positioned at vertices of the tetragonal UF_2_O_4_ bipyramid. The U2 site (Wyckoff notation 4*b*, site symmetry 1) is surrounded by seven ligands, including two axial uranyl O atoms (O1 and O5) and five equatorial ligands (O2 2×; O6 2×; F1), forming a UFO_6_ pentagonal bipyramid (Fig. 6 ▸). The bond lengths of the U1—F bonds are 2.19 and 2.21 Å (Table 3 ▸) and the bond-valence sums around the U1 site (Table 4 ▸) are consistent with this U being pentavalent. The uranyl pentagonal bipyramids share edges to form chains along [100] and chains of edge-sharing squares (occupied by UF_4_O_2_) and triangles along the same direction (Fig. 7 ▸ *a*); this results in the sheets stacked perpendicular to [001], which belong to the β-U_3_O_8_ topology (Fig. 7 ▸ *b*) (Burns, 2005 ▸). The Mg1 site is octahedrally coordinated by two mutually trans F atoms (belonging to the U1 site) and four O atoms in equatorial configuration (two O3 atoms related by symmetry, O4 and O7 atoms) that are H_2_O molecules. According to the results of the electron microprobe analyses, nollmotzite contains minor Cu along with dominant Mg. A partial substitution of Cu at the Mg site was documented by the site-scattering refinement; however, because of the small amount of Cu^2+^ entering the site (less than 0.2 atoms per unit cell), no distortion of the octahedral coordination due to the Jahn–Teller effect was observed (see polyhedral distortion parameters for the Mg polyhedron; Table 3 ▸). Adjacent sheets of U polyhedra are linked through the F—Mg—F linkages, as well as through hydrogen bonds. The arrangement of the hydrogen bonds network can be deduced based on the bond-valence analysis (Table 4 ▸). Within the structural sheets the only acceptors of the hydrogen bonds, which emanate from the H_2_O groups (O3, O5, O4, O7 coordinated to Mg1/Cu1 atom) in interlayer, are uranyl oxygen atoms (O1 and O5) linked to the U2 atom. The O⋯O distances related to hydrogen-bond interactions are 2.81 (3) Å (O3⋯O1), 2.83 (3) Å (O3⋯O5), 2.89 (3) Å (O4⋯O1), and 2.90 (4) Å (O7⋯O5). The respective angles (acceptor—donor—acceptor), O1—O4—O1 (101.8°), O5—O7—O5 (102.4°) and O1—O3—O5 (112.2°), are similar to the theoretical H—O—H angle (104.5°) in an H_2_O molecule.

The structural formula of nollmotzite obtained from the refinement and bond-valence considerations is {^[6]^Mg(H_2_^[3]^O)_4_}^2+^[U^V^(U^VI^O_2_)_2_F_3_O_4_]^2–^, *Z* = 2.

## 4. Discussion   

### 4.1. Nollmotzite and related minerals and compounds containing U^V^   

Nollmotzite, Mg[U^V^(U^VI^O_2_)_2_F_3_O_4_](H_2_O)_4_, is the fourth mineral known to contain pentavalent uranium, the others being wyartite, Ca(CO_3_)[U^V^(U^VI^O_2_)_2_O_4_(OH)](H_2_O)_7_ (Burns & Finch, 1999 ▸), shinkolobweite, Pb_1.25_[U^V^(H_2_O)_2_(U^VI^O_2_)_5_O_8_(OH)_2_](H_2_O)_5_ (Olds *et al.*, 2017 ▸), and richetite, Fe_0.5_Pb_5_[U^V^(U^VI^O_2_)_17_O_18_(OH)_14_](H_2_O)_∼19.5_ (Plášil, 2017 ▸). Dehydrated wyartite, Ca(CO_3_)[U^V^(U^VI^O_2_)_2_O_4_(OH)](H_2_O)_3_ (Hawthorne *et al.*, 2006 ▸), which also contains U^V^, is not approved officially as a mineral by the International Mineralogical Association. While nollmotzite, both hydrated and dehydrated wyartite, and shinkolobweite contain structural sheets based on β-U_3_O_8_ topology, richetite possesses sheets of α-U_3_O_8_ topology (the fourmarierite type). The family of synthetic compounds that contains U^V^ is broader. Among them, the most similar to the aforementioned mineral structures is that of synthetic [U^V^(H_2_O)_2_(U^VI^O_2_)_2_O_4_(OH)](H_2_O)_4_ (Belai *et al.*, 2008 ▸), whose structure is also based upon sheets of β-U_3_O_8_ topology.

The incorporation of fluorine into uranyl oxide sheet structures has not been previously observed, but the nollmotzite structure demonstrates that it is possible. Both α-U_3_O_8_ and β-U_3_O_8_ topologies have the same U:O ratio (3:5), and also similar U:OH content (Krivovichev, 2013 ▸; Plášil, 2018 ▸). We can expect that the incorporation of fluorine is of equal probability for both topological types.

Recently, two synthetic phases, uranyl oxides that contain fluorine, have been synthesized. The structure of synthetic phase [(UO_2_)_4_F_13_][Sr_3_(H_2_O)_8_](NO_3_)·H_2_O (Jouffret *et al.*, 2016 ▸) is based upon sheets, where fluorine acts as a ligand of pentagonal bipyramids coordinating U^VI^. Felder *et al.* (2018 ▸) synthesized a mixed Co^II^–uranyl–oxide–fluoride hexahydrate, [Co(H_2_O)_6_]_3_[U_2_O_4_F_7_]_2_. Nevertheless, this synthetic phase contains only hexavalent U. Thus, fluorine acts as an equatorial ligand of UO_2_^2+^. The structure is based upon infinite chains of [U_2_O_4_F_7_] dimer units.

### 4.2. Remarks on the coordination of U^V^ and U^VI^ in the solid state   

The U^VI^, present as the uranyl ion UO_2_^2+^ (UrO_2_), occurs most typically in three types of coordination polyhedra: (1) square bipyramids (UrO_2_Φ_4_; four equatorial ligands), (2) pentagonal bipyramids (UrO_2_Φ_5_; five equatorial ligands), and (3) hexagonal bipyramids (UrO_2_Φ_6_; six equatorial ligands). A bimodal distribution of bond lengths is observed for uranyl pentagonal and hexagonal bipyramids. For both, the U—O_yl_ bond lengths in the uranyl ion are significantly shorter than the U—O_eq_ bonds (Burns *et al.*, 1997*a* ▸; Lussier *et al.*, 2016 ▸; following values are based upon 222 well refined structures). The average ^[7]^U^6+^—O_yl_ is 1.793 Å (σ = 0.035 Å); ^[7]^U^6+^—O_eq_ is 2.368 Å (σ = 0.100 Å). The average ^[8]^U^6+^—O_yl_ bond is 1.783 (σ = 0.030 Å); the average U^6+^—O_eq_ bond is 2.460 Å (σ = 0.107 Å). In the case of ^[6]^U^6+^, the uranyl cation is coordinated by four equatorial ligands with an average U—O_yl_ bond of 1.816 Å (σ = 0.050 Å) and an average U—O_eq_ bond of 2.264 (σ = 0.064 Å). Nevertheless, there are also examples of structures containing U^6+^ in a regular (or distorted) octahedral coordination, where the average U—O bond lengths are ∼2.1 Å (*e.g.* Morrison *et al.*, 2011 ▸). Furthermore, in at least two reported structures (Wu *et al.*, 2009 ▸; Unruh *et al.*, 2010 ▸), ^[6]^U^6+^ adopts an unusual tetraoxido core, wherein the four equatorial bonds of the octahedra are short, ∼1.8 Å, and the axial bonds are longer, ∼2.3 Å.

In contrast to U^VI^ compounds, there are only a few well defined U^V^ structures known. Among the oxo-compounds, the most common coordination number of U^V^ is [7] (Table S4); there are 56 individual values with an average U^V^—O bond length of 2.25 Å (σ = 0.03 Å) (with the median at 2.20 Å); the distribution of bond lengths (Fig. S1) shows a positive skewness (1.335).

**Figure 8:**
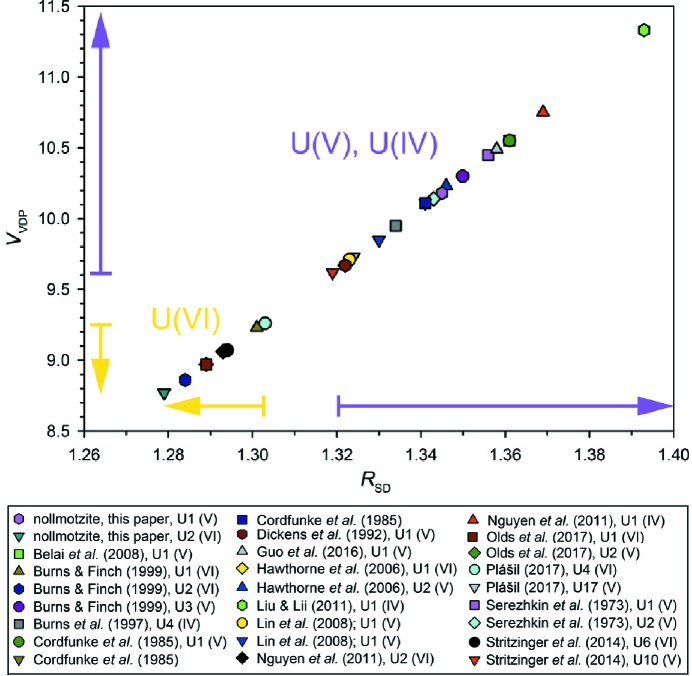
A plot of Voroni–Dirichlet polyhedra (VDP) measures: equivalent radius of the sphere (*R*_SD_, in Å) and a volume of VDP (*V*_VDP_, in Å^3^) displaying characteristic values for U^VI^, U^V^ and U^IV^ VDP as well.

To probe the character of bonding interactions within the U^V^–Φ polyhedra, an analysis using Voronoi–Dirichlet polyhedra (VDP) was carried out, the results of which are given in Table 5 ▸. The values of the second momentum of inertia, *G*_3_, which characterizes the sphericity of VDP, ranges from 0.082 to 0.089. The *G*_3_ value of 0.082 is taken as the threshold indicating covalent bonding character (Blatov & Serezhkin, 2000 ▸). An interesting feature that distinguishes U^V^ from U^VI^ is the volume of corresponding VDP, and consequently also the equivalent radius, *R*_SD_. The relationship displayed in Fig. 8 ▸, showing a linear trend, allows U^V^ (higher VDP) to be distinguished from U^VI^ (lower VDP). The lowest VDP volume observed for U^V^ is 9.62 Å^3^ (with equivalent radius, *R*_SD_, of 1.32 Å), while the largest VDP volume observed for U^VI^ is 9.26 Å^3^ (with *R*_SD_ = 1.30 Å).

## 5. Implications   

The formation of nollmotzite is a result of solid-state precipitation from U-containing aqueous solutions under partially reducing conditions. Nollmotzite contains ∼20 mol.% F in a structural unit, suggesting that the fugacity of O_2_ was lower than under fully oxidizing conditions. The reducing environment was most probably enhanced by consumption of oxygen during the oxidation of abundant pyrite present in the gangue (crusts of Fe^III^ oxy­hydroxides are abundant on the samples). The major gangue minerals are dark varieties of fluorite (called *stinkspath* or *antozonite*), barite and quartz. The high fugacity of fluorine and low fugacity of oxygen led to the incorporation of fluorine into the sheet structure of nollmotzite and a partial reduction of U^VI^ to U^V^. The discovery of nollmotzite contributes to the large body of radioactive-waste final-disposal research. In designing final repositories for radioactive waste, the paradigm has shifted from using oxidative conditions towards using strongly reducing conditions (Ewing, 2015 ▸; Ewing *et al.*, 2016 ▸; Rojo *et al.*, 2018 ▸). Reducing environments are considered preferable to prevent the corrosion of the stainless steel tanks embedded in clay/cement after the closure of the repository. In spite of efforts to create overall reducing conditions, oxygen atoms will continue to be available from groundwater and/or dissolved oxygen-containing minerals in the backfill. Stainless steel used for the fabrication of tanks in the repository generally does not contain fluorine (even if there are some industrial passivation processes based on fluorination). However, traces of fluorine can be derived from dissolution of phosphate minerals present in the backfill, or it can be present in the groundwater. The corrosion processes take place at the interfaces between radioactive waste and tanks/backfill/surrounding rocks, and they are often bounded onto small (micro) areas with strong geochemical gradients.

## 6. Conclusions   

The new mineral found at the Clara mine, Black Forest Mountains, Germany, is the first known naturally occurring uranium oxide that contains significant fluorine. Furthermore, the reducing conditions under which this mineral formed led to the partial reduction of the U^VI^ to U^V^. Therefore, nollmotzite is one of the very few minerals containing pentavalent uranium. The characterization of new supergene uranium minerals with unusual structural and/or compositional features, such as nollmotzite, can provide valuable insights into processes that may occur during the long-term storage of spent nuclear fuel.

## Supplementary Material

Crystal structure: contains datablock(s) global, I. DOI: 10.1107/S2052520618007321/lo5026sup1.cif

Structure factors: contains datablock(s) I. DOI: 10.1107/S2052520618007321/lo5026Isup2.hkl

Supplementary tables S1, S2, S3, S4 and Fig. S1. DOI: 10.1107/S2052520618007321/lo5026sup3.pdf

CCDC reference: 1843381

## Figures and Tables

**Figure 2:**
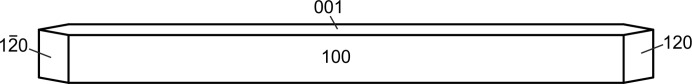
Crystal drawing of nollmotzite; clinographic projection in standard orientation.

**Table 1:** Chemical composition (in wt%) of nollmotzite

Constituent	Mean of six spots (wt%)	Range	Standard deviation	Theoretical composition†	Standard
MgO	4.20	4.03–4.37	0.13	4.05	Mg_2_SiO_4_
CuO	0.12	0.05–0.35	0.11		Lammerite
UO_3_	(84.18)	83.28–85.28	0.58	(86.20)	Parsonsite
U_2_O_5_ ‡	27.28			27.93	
UO_3_ ‡	56.12			57.47	
F	5.87	5.39–6.16	0.25	5.72	Topaz
H_2_O§	6.80			7.24	
—O=F	−2.47			−2.41	
Total	97.92			100.00	

† Mg[U^V^(U^VI^O_2_)_2_F_3_O_4_](H_2_O)_4_.

‡ Apportioned in accord to the structure.

§ Based on structure.

**Table 2:** Data collection and refinement details for nollmotzite

Chemical formula	(Mg_0.82_Cu_0.18_)_Σ1.00_[U^V^(U^VI^O_2_)_2_F_3_O_4_](H_2_O)_4_
*M*_r_	1002.66
Space group	*Cm*
*a*, *b*, *c* (Å)	7.1015 (12), 11.7489 (17), 8.1954 (14)
β (°)	98.087 (14)
*V* (Å^3^)	676.98 (19)
*Z*	2
No. of reflections for unit-cell parameters	695
θ range (°)	3.38–29.34
μ (mm^−1^)	36.20
Crystal form, size (mm)	Prismatic, 0.072 × 0.012 × 0.009
	
Wavelength (Å)	0.71073
Temperature (K)	296
Absorption correction	Multi-scan with empirical absorption correction using spherical harmonics, implemented in SCALE3 ABSPACK scaling algorithm
*T*_min_, *T*_max_	0.622, 1
No. of measured, independent and observed [*I* > 3σ(*I*)] reflections	3100, 2022, 1527
*R*_int_	0.027
(sinθ/λ)max (Å^−1^)	0.689
	
Refinement on *F* ^2^	
*R*_obs_, *R*_all_	0.0369, 0.0757
*wR*_obs_, *wR*_all_	0.0551, 0.0880
*S* (all)	1.09
Δρ_min_, Δρ_max_ (e Å^−3^)	−2.28, 2.74
Twin matrix	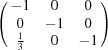
Twvol_1_, twvol_2_	0.5892 (12), 0.4108 (12)

Computer programs: *CrysAlis PRO* v.1.171.38.43 (Rigaku Oxford Diffraction, 2017 ▸), *JANA2006* (Petříček *et al.*, 2014 ▸).

**Table 3:** Selected interatomic distances (in Å) and measures of polyhedral distortion parameters for nollmotzite Φ and Φ_eq_ denote ligands and equatorial ligands, respectively. O_Ur_ denotes uranyl O atoms. ECoN is effective coordination number (Hoppe, 1979 ▸); octahedral distortion (Robinson *et al.*, 1971 ▸); bond-angle variance (deg^2^) (Robinson *et al.*, 1971 ▸). Calculations of polyhedral distortion measured using *VESTA* (Momma & Izumi, 2011 ▸) software.

U1—O2	2.08 (2)	U2—O1	1.807 (18)	Mg1—F2^iv^	1.92 (4)
U1—O2^i^	2.08 (2)	U2—O5	1.77 (2)	Mg1—O3	2.061 (12)
U1—O6	2.16 (2)	U2—O2	2.34 (2)	Mg1—O3^i^	2.061 (12)
U1—O6^i^	2.16 (2)	U2—O2^ii^	2.36 (2)	Mg1—O4	2.07 (4)
U1—F2	2.19 (3)	U2—O6^ii^	2.31 (2)	Mg1—F3	1.99 (4)
U1—F3	2.21 (3)	U2—O6^iii^	2.30 (2)	Mg1—O7	2.20 (5)
〈U1—Φ〉	2.15	U2—F1	2.289 (3)	〈Mg1—Φ〉	2.05
		〈U2—O_Ur_〉	1.79	*V*_Mg_ (Å^3^)	11.41
		〈U2—Φ_eq_〉	2.32	ECoN	5.53
				Distortion	0.031
				Bond-angle variance	14.06

Symmetry codes: (i) *x*, −*y* + 1, *z*; (ii) *x* − 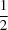, −*y* + 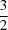, *z*; (iii) *x* − 1, *y*, *z*; (iv) *x*, *y*, *z* − 1.

**Table 4:** Bond-valence analysis (all values given in valence units, vu) of the crystal structure of nollmotzite The bond-valence parameters are taken from Brown & Altermatt (1985 ▸) (for Mg—F), Brese & O’Keeffe (1991 ▸) (for Cu—O), Burns *et al.* (1997*a* ▸) (for U^VI^—O), Gagné & Hawthorne (2015 ▸) (for Mg—O) and Zachariasen (1978 ▸) (for U^V^—F and U^VI^—F). Bond strengths of hydrogen bonds are taken from Hawthorne & Schindler (2008 ▸) and correspond to an average *D*—H bond strength (0.8 vu) and an average H⋯*A* bond strength (0.2 vu). ↓ and → denote the direction in which multiplication should be applied.

Atom	U1	U2	Mg1*/Cu1*	ΣBV	Assignment
O1		1.59		1.59	O^2−^ (+2 × 0.2 vu)
O2	0.99 × 2↓	0.56, 0.54		2.09	O^2–^
O3			0.72	0.72	H_2_O (+2 × 0.8 vu)
O4			0.35	0.35	H_2_O (+2 × 0.8 vu)
O5		1.71		1.71	O^2−^ (+2 × 0.2 vu)
O6	0.86 × 2↓	0.59, 0.61		2.06	O^2−^
O7			0.26	0.26	H_2_O (+2 × 0.8 vu)
F1		0.46 × 2→		0.92	F^−^
F2	0.59		0.41	1.00	F^−^
F3	0.56		0.34	0.90	F^−^
ΣBV	4.85	6.07	2.07		

**Table 5:** Voronoi–Dirichlet polyhedra parameters for U atoms in the selected structures

Structure; #atom	U*^n^*	CN	*V*_VDP_ (Å^3^)	*R*_SD_ (Å)	*G*_3_	Ref.
Nollmotzite; U1	5	6	10.1800	1.3450	0.0852	(*a*)
Nollmotzite; U2	6	7	8.7700	1.2790	0.0833	
Richetite; U17	5	(6)	9.7300	1.3240	0.0850	(*b*)
Richetite; U4	6	7	9.2600	1.3030	0.0855	
Wyartite; U1	6	7	9.2300	1.3010	0.0832	(*c*)
Wyartite; U2	6	7	8.8600	1.2840	0.0835	
Wyartite; U3	5	7	10.3000	1.3499	0.0837	
Dehydr. wyartite; U1	6	7	8.9700	1.2890	0.0831	(*d*)
Dehydr. wyartite; U2	5	7	10.2300	1.3460	0.0840	
Shinkolobweite; U1	6	7	8.9700	1.2890	0.0836	(*e*)
Shinkolobweite; U2	5	6	9.6700	1.3220	0.0889	
Ianthinite; U4	4	6	9.9500	1.3340	0.0851	(*f*)
[U^V^(H_2_O)_2_(U^VI^O_2_)_2_O_4_(OH)](H_2_O)_4_; U1	5	6	10.5500	1.3610	0.0857	(*g*)
K_13_[(UO_2_)_19_(UO_4_)(B_2_O_5_)_2_(BO_3_)_6_(OH)_2_O_5_](H_2_O); U6	6	7	9.0700	1.2940	0.0836	(*h*)
K_13_[(UO_2_)_19_(UO_4_)(B_2_O_5_)_2_(BO_3_)_6_(OH)_2_O_5_](H_2_O); U10	5	6	9.6200	1.3190	0.0872	
Cs_8_U^IV^(U^VI^O_2_)_3_(Ge_3_O_9_)_3_(H_2_O)_3_; U1	4	6	10.7500	1.3690	0.0833	(*i*)
Cs_8_U^IV^(U^VI^O_2_)_3_(Ge_3_O_9_)_3_(H_2_O)_3_; U2	6	6	9.0600	1.2930	0.0850	
Cs_2_U^IV^Si_6_O_15_; U1	4	6	11.3300	1.3930	0.08374	(*j*)
K_3_(U^V^_3_O_6_)(Si_2_O_7_); U1	5	6	9.7100	1.3230	0.08354	(*k*)
U_2_MoO_8_; U1	5	7	10.4500	1.3560	0.08350	(*l*)
U_2_MoO_8_; U2	5	7	10.1400	1.3430	0.08340	
UTa_3_O_10_; U1	5	8	10.4900	1.3580	0.08316	(*m*)
UVO_5_; U1	5	7	9.6700	1.3220	0.08201	(*n*)
U_5_O_12_Cl; U1	5	6	10.5500	1.361	0.08498	(*o*)
U_5_O_12_Cl; U2	5	7	10.1100	1.341	0.08275	
U_5_O_12_Cl; U3	5	7	10.1100	1.341	0.08212	

U*^n^* is valence state; CN is coordination number; *V*_VDP_ is volume of the Voronoi–Dirichlet polyhedron; *R*_SD_ is radius of the sphere with volume equal to that of the VDP; *G*_3_ is the second moment of inertia (approximately, the deviation of the VD from ideal sphere). References: (*a*) this work, (*b*) Plášil (2017 ▸), (*c*) Burns & Finch (1999 ▸), (*d*) Hawthorne *et al.* (2006 ▸), (*e*) Olds *et al.* (2017 ▸), (*f*) Burns *et al.* (1997*b* ▸), (*g*) Belai *et al.* (2008 ▸), (*h*) Stritzinger *et al.* (2014 ▸), (*i*) Nguyen *et al.* (2011 ▸), (*j*) Liu & Lii (2011 ▸), (*k*) Lin *et al.* (2008 ▸), (*l*) Serezhkin *et al.* (1973 ▸), (*m*) Guo *et al.* (2016 ▸), (*n*) Dickens *et al.* (1992 ▸), (*o*) Cordfunke *et al.* (1985 ▸).
