# Supplementary material for: Nollmotzite, Mg[UV(UVIO2)2O4F3]·4H2O, the first natural uranium oxide containing fluorine
Source: Acta Crystallogr B Struct Sci Cryst Eng Mater. 2018 Jun 28;74(Pt 4):362–9. doi: 10.1107/S2052520618007321 (PMC6108157; doi:10.1107/S2052520618007321)
Supplement: Supplementary file 3 [file b-74-00362-sup3.pdf]

STRUCTURAL SCIENCE
CRYSTAL ENGINEERING
MATERIALS

Volume 74 (2018)

Supporting information for article:

Nollmotzite, $Mg[U^{V}(U^{VI}O2)2O4F3]·4H2O$, the first natural uranium-oxide containing fluorine

Jakub Plášil, Anthony R. Kampf, Radek Škoda and Jiří Čejka

Table S1

Powder X-ray data (*d* in Å) for nollmotzite. Only calculated lines with $I_{\mathrm{rel.}}$>1 are shown.

I_{obs}	d_{obs}	d_{calc}	I_{calc}	*hkl*
**100**	**8.10**	8.1207	100	0 0-1
6	6.02	6.0470	4	-1-1 0
7	4.760	4.7695	4	0-2-1
**31**	**4.060**	4.0603	26	0 0-2
**30**	**3.518**	3.5224	20	-2 0 0
**54**	**3.420**	3.4312	31	-1-3 0
		3.4128	14	2 0-1
**22**	**3.237**	3.2418	23	1-3-1
**26**	**3.083**	3.0852	18	-1-3-1
		3.0763	7	-2 0-1
3	2.954	2.9465	3	0-4 0
7	2.868	2.8695	7	2 0-2
		2.7698	2	0-4-1
**17**	**2.710**	2.7147	9	1-3-2
		2.7069	8	0 0-3
5	2.533	2.5359	5	-1-3-2
5	2.492	2.4917	5	-2 0-2
		2.3847	2	0-4-2
3	2.307	2.3103	3	2 0-3
6	2.248	2.2600	4	-2-4 0
		2.2354	2	-1-5 0
5	2.214	2.2303	4	2-4-1
		2.2003	5	1-3-3
5	2.182	2.1804	3	1-5-1
5	2.126	2.1308	2	-1-5-1
		2.1279	2	-2-4-1
9	2.0525	2.0573	6	-1-3-3
		2.0557	2	2-4-2
		2.0302	6	0 0-4
		2.0157	8	-3-3 0
**32**	**2.0148**	2.0144	8	3-3-1
		2.0130	3	-2 0-3
5	1.9603	1.9643	4	0-6 0
		1.9093	3	0-6-1
16	1.9028	1.9029	6	-3-3-1
		1.8998	5	3-3-2
4	1.8793	1.8778	4	2 0-4
5	1.8033	1.8026	5	1-3-4
		1.7740	2	4 0-1
11	1.7653	1.7683	3	0-6-2
		1.7612	3	-4 0 0
		1.7239	4	-3-3-2
13	1.7179	1.7200	4	3-3-3
		1.7156	3	-2-6 0
		1.7064	2	4 0-2
8	1.6980	1.7025	2	2-6-1
		1.6967	4	-1-3-4
8	1.6638	1.6601	3	-2 0-4
4	1.6373	1.6376	4	-1-7 0
		1.6241	2	0 0-5
5	1.6186	1.6209	2	2-6-2
		1.6156	3	1-7-1
6	1.5886	1.5951	2	-1-7-1
		1.5898	2	0-6-3
		1.5426	2	-2-6-2
6	1.5348	1.5382	2	-4 0-2
		1.5364	2	1-7-2
5	1.5256	1.5300	3	-3-3-3
		1.5262	3	3-3-4
3	1.5064	1.5088	3	1-3-5
		1.5017	2	-1-7-2

Table S2 Atomic coordinates, occupational and equivalent displacement parameters (Å^2) for the structure of nollmotzite.

Atom	*x/a*	*y/b*	*z/c*	U_{eq}	*Occupancy*
U1	0.5007(4)	0.5	0.8023(5)	0.0093(3)	
U2	0.0010(4)	0.69427(4)	0.7998(4)	0.00740(18)	
Mg1	0.506(3)	0.5	0.302(4)	0.014(2)	0.816(16)
Cu1	0.506(3)	0.5	0.302(4)	0.014(2)	0.184(16)
O1	0.049(3)	0.691(2)	1.022(2)	0.017(6)*	
O2	0.317(3)	0.6380(18)	0.796(3)	0.0074(16)*	
F1	0.001(5)	0.5	0.820(4)	0.023(2)*	
F2	0.533(3)	0.5	1.072(4)	0.023(2)*	
O3	0.529(3)	0.6749(10)	0.304(3)	0.023(4)*	
O4	0.219(5)	0.5	0.211(5)	0.028(8)*	
O5	−0.023(3)	0.692(2)	0.582(3)	0.013(5)*	
F3	0.438(3)	0.5	0.530(4)	0.023(2)*	
O6	0.685(3)	0.6450(18)	0.793(3)	0.0074(16)*	
O7	0.813(6)	0.5	0.391(7)	0.053(13)*	

Table S3 Anisotropic displacement parameters (Å^2) for the structure of nollmotzite.

Atom	U^{11}	U^{22}	U^{33}	U^{12}	U^{13}	U^{23}
U1	0.0091(3)	0.0043(4)	0.0149(6)	0	0.0027(3)	0
U2	0.0055(2)	0.0013(2)	0.0155(4)	0.0000(13)	0.0020(2)	−0.0034(13)
Mg1/Cu1	0.026(3)	0.007(3)	0.006(4)	0	−0.004(3)	0

Table S4 Bond-lengths in coordination polyhedra around U(V) atoms in selected compounds and their polyhedral measures.

Structure; #atom	U–Φ (in Å)								Ref.
Nollmotzite; U1	2.08	2.08	2.16	2.16	2.19	2.21			[1]
Richetite; U17	2.16	2.94	2.17	2.23	2.01	2.01	2.19		[2]
Wyartite; U3	2.07	2.09	2.06	2.14	2.44	2.47	2.48		[3]
Dehydr. wyartite; U2	2.10	2.10	2.09	2.09	2.48	2.51	2.30		[4]
Shinkolobweite; U2	2.25	2.25	2.13	2.13	1.99	1.96			[5]
$[U^{V}(H_2O)_2(U^{VI}O_2)_2O_4(OH)](H_2O)_4$; U1	2.06	2.06	2.06	2.06	2.41	2.41			[6]
$K_{13}[(UO_2)_{19}(UO_4)(B_2O_5)_2(BO_3)_6(OH)_2O_5](H_2O)$; U10	1.81	1.81	2.17	2.17	2.38	2.38			[7]
$K_3(U^{V}{}_3O_6)(Si_2O_7)$; U1	2.09	2.09	2.12	2.12	2.19	2.19			[8]
U_2MoO_8; U1	2.06	2.06	2.11	2.18	2.36	2.46	2.73		[9]
U_2MoO_8; U2	2.08	2.08	2.13	2.15	2.32	2.35	2.58		
UTa_3O_{10}; U1	1.85	1.85	2.55	2.55	2.55	2.55	2.59	2.59	[10]

UVO_5; U1	2.05	2.08	2.21	2.21	2.30	2.33	2.30	[11]
$U_5O_{12}Cl$; U1	2.07	2.07	2.14	2.33	2.27	2.19		[12]
$U_5O_{12}Cl$; U2	2.07	2.07	2.23	2.30	2.16	2.27	2.70	
$U_5O_{12}Cl$; U3	2.07	2.07	2.30	2.25	2.25	2.30	2.54	

References: [1] this work, [2] Plášil (2017), [3] Burns & Finch (1999), [4] Hawthorne *et al.* (2006), [5] Olds *et al.* (2017), [6] Belai et al. (2008), [7] Stritzinger *et al.* (2014), [8] Lin *et al.* (2008), [9] Serezhkin *et al.* (1973), [10] Guo *et al.* (2016), [11] Dickens et al. (1992), [12] Cordfunke *et al.* (1985).

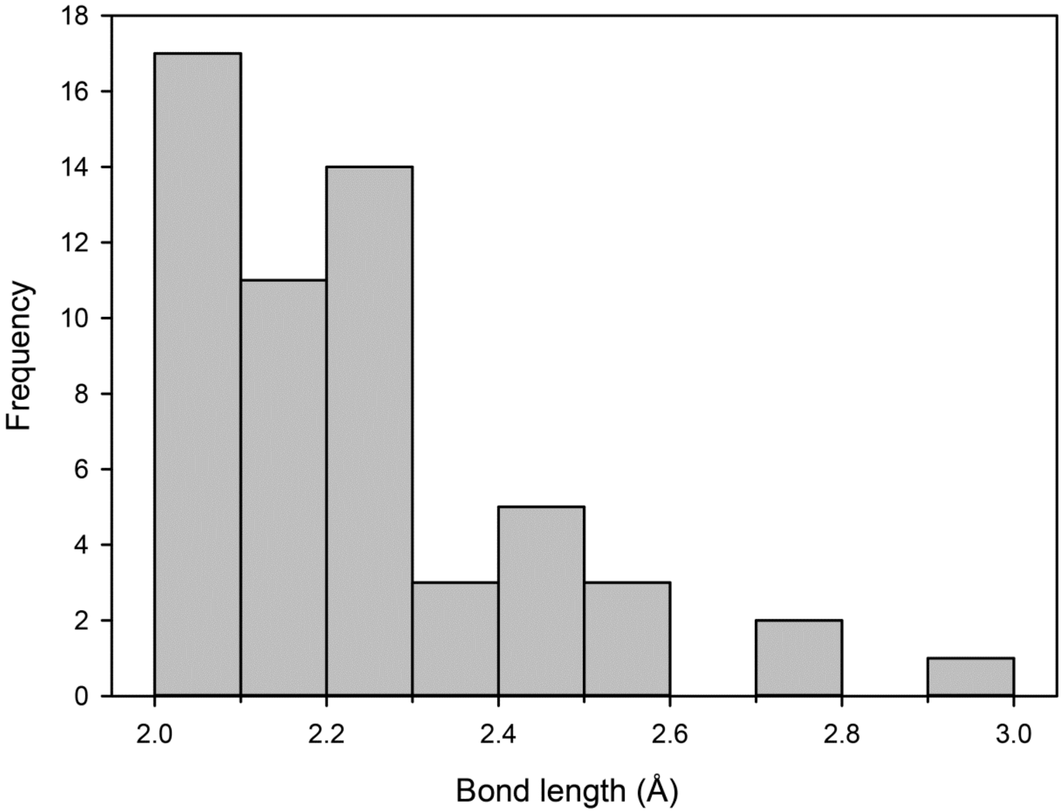

Figure S1 Bond-length distribution for U(V)–Φ bonds.
